# Activation of GSK3β by Sirt2 Is Required for Early Lineage Commitment of Mouse Embryonic Stem Cell

**DOI:** 10.1371/journal.pone.0076699

**Published:** 2013-10-18

**Authors:** Xiaoxing Si, Wen Chen, Xudong Guo, Long Chen, Guiying Wang, Yanxin Xu, Jiuhong Kang

**Affiliations:** Clinical and Translational Research Center of Shanghai First Maternity & Infant Health Hospital, Shanghai Key Laboratory of Signaling and Disease Research, School of Life Science and Technology, Tongji University, Shanghai, China; National University of Singapore, Singapore

## Abstract

Sirt2, a member of the NAD^+^-dependent protein deacetylase family, is increasingly recognized as a critical regulator of the cell cycle, cellular necrosis and cytoskeleton organization. However, its role in embryonic stem cells (ESCs) remains unclear. Here we demonstrate that Sirt2 is up-regulated during RA (retinoic acid)-induced and embryoid body (EB) differentiation of mouse ESCs. Using lentivirus-mediated shRNA methods, we found that knockdown of Sirt2 compromises the differentiation of mouse ESCs into ectoderm while promoting mesoderm and endoderm differentiation. Knockdown of Sirt2 expression also leads to the activation of GSK3β through decreased phosphorylation of the serine at position 9 (Ser9) but not tyrosine at position 216 (Tyr216). Moreover, the constitutive activation of GSK3β during EB differentiation mimics the effect of Sirt2 knockdown, while down-regulation of GSK3β rescues the effect of Sirt2 knockdown on differentiation. In contrast to the effect on lineage differentiation, Sirt2 knockdown and GSK3β up-regulation do not change the self-renewal state of mouse ESCs. Overall, our report reveals a new function for Sirt2 in regulating the proper lineage commitment of mouse ESCs.

## Introduction

Embryonic stem cells (ESCs) are pluripotent and self-renewing cells that are derived from the inner cell mass of an embryo [1]. In vivo, ESCs have the ability to generate all three germ layers: ectoderm, mesoderm, and endoderm. In vitro, if cultured in specific conditions, ESCs can differentiate into multiple cell lineages [2]. These unique properties make them especially valuable for regenerative medicine and cell replacement therapies. However, in vitro induction of ESC differentiation into a particular cell lineage often results in only a small proportion of properly differentiated cells [3]. Thus, understanding the mechanism of lineage commitment is necessary for the development of practical applications for ESCs.

Recent studies have uncovered several signaling pathways involved in regulating the pluripotency of mouse ESCs, of which the Wnt/β-catenin signaling pathway has been shown to play a critical role. Although studies disagree on whether it functions to promote or inhibit stemness in human ESCs [4]–[6], it is widely believed that the Wnt/β-catenin signaling pathway promotes pluripotency and self-renewal in mouse ESCs. GSK3β, a negative regulator of the Wnt/β-catenin signaling pathway, has recently been implicated in a diverse array of cellular functions, including embryonic stem cell pluripotency. GSK3β is a multifunctional serine/threonine (Ser/Thr) kinase found ubiquitously in eukaryotes [5],[7]. Phosphorylation of the serine at position 9 (Ser9) inhibits its activity, whereas phosphorylation of the tyrosine at position 216 (Tyr216) increases it. Inhibition of GSK3β using the specific inhibitors BIO [8] or CHIRON99021 [5] is sufficient to support the short-term expansion of mouse ESCs [8]–[9]. The effect of GSK3β inhibition on ESC self-renewal is mediated primarily via phosphorylation of β-catenin because ESCs lacking β-catenin do not respond productively to GSK3β inhibitors. Based on studies performed in ESCs derived from embryos deleted for GSK3β, it is clear that β-catenin-independent effects are critically important and represent the canonical pathway in the differentiation of mouse ESCs. Most studies have focused on the role and mechanism of GSK3β in controlling ESC pluripotency, but the understanding of the role of upstream modulators of GSK3β in regulating self-renewal and differentiation is limited.

The mammalian sirtuin family comprises seven members, Sirt1–Sirt7, which are homologous to yeast Sir2. Sirtuins have been reported to regulate the cell cycle, gene silencing, apoptosis and energy homeostasis [10]. Sirt1 is the most well studied mammalian sirtuin and participates in heterochromatin formation, cell growth and ESC differentiation [11]–[13]. Sirt1 is involved in lineage specification in both human and mouse ESCs [14]. Unlike Sirt1, the function of Sirt2 is not well understood. Recent studies revealed that the lack of Sirt2 can block cellular necrosis induced by TNF-α. In mammalian cells, Sirt2 has been shown to be involved in the cell cycle and cytoskeleton organization [15]. Down-regulation of Sirt2 promotes 3T3L1 adipocyte differentiation due to increased acetylation and phosphorylation of Foxo1 and its consequent translocation to the cytoplasm [16]. Although a previous report shows that knockdown or inhibition of Sirt2 with AC93253 in myeloid leukemia cells activates GSK3β through decreased Ser9 phosphorylation and prevents aberrant proliferation [10], little is known about the role of Sirt2 and GSK3β in ESCs. Our studies demonstrate that Sirt2 is up-regulated during the differentiation of mouse ESCs. Sirt2 knockdown results in the promotion of mesoderm and endoderm differentiation and compromises the differentiation of ectoderm; these effects are mediated through the activation of GSK3β.

## Results

### Sirt2 is Up-regulated during both RA-mediated and EB Differentiation of Mouse ESCs

**Figure 1:**
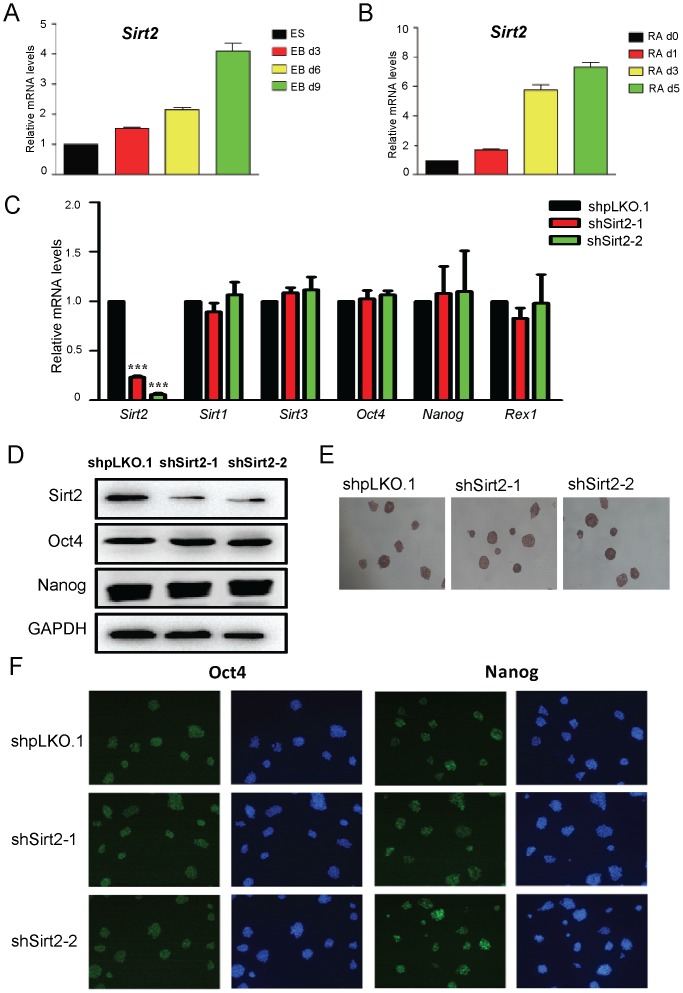
Up-regulation of Sirt2 during mouse ESC differentiation. (**A**) Real-time PCR analysis of Sirt2 in EB differentiation. (**B**) The mRNA expression level of Sirt2 during RA-induced differentiation. (**C**) The mRNA expression levels of Sirt2, Sirt1, Sirt3 and self-renewal marker genes Oct4, Nanog and Rex1 in control (shpLKO.1) and Sirt2 knockdown cell lines (shSirt2-1 and shSirt2-2). Data were normalized to *Gapdh* mRNA expression levels and are means ±SEM (n = 3). *P*<0.001(***) vs. shpLKO.1 cell lines (One-way ANOVA). (**D**) Western blots analyzing Sirt2, Oct4 and Nanog levels in shpLKO.1, shSirt2-1 and shSirt2-2 mouse ESCs. GAPDH was used as a protein loading control. (**E**) AP staining of control and two Sirt2 knockdown cell lines. All Figures 100×. (**F**) Immunofluorescence staining of self-renewal marker genes Oct4 and Nanog in control and two Sirt2 knockdown cells. All Figures 100×.

To investigate the role of Sirt2 in mouse ESCs, we assessed the expression levels of Sirt2 during mouse ESC differentiation (Fig. 1A, B). Real-time PCR analysis showed that Sirt2 expression was up-regulated in both RA-mediated and EB differentiation of mouse ESCs, indicating that Sirt2 may have a role in mouse ESC differentiation. We then examined the effect of knocking down Sirt2 expression in mouse ESCs. We knocked down Sirt2 expression with two independent lentivirus-mediated shRNAs (shSirt2-1 and shSirt2-2). Sirt2 mRNA levels were successfully depleted up to 77% and 94% with shSirt2-1 and shSirt2-2 in mouse ESCs, respectively (Fig. 1C). Sirt1 and Sirt3 levels were unchanged in the context of Sirt2 knockdown, which confirmed the specificity of the shRNAs (Fig. 1C). The protein levels of Sirt2 were examined by Western blot analysis, which also demonstrated dramatic knockdown (Fig. 1D). Sirt2 depletion did not affect the mRNA expression levels of the known ES cell self-renewal genes, Oct4, Nanog and Rex1 (Fig. 1C). Using Western blot analysis, the protein levels of Nanog and Oct4 in ESCs upon efficient Sirt2 knockdown remained unchanged (Fig. 1D), which was consistent with immunostaining analysis (Fig. 1F). Sirt2 knockdown cells were also able to form alkaline phosphatase positive colonies as efficiently as control ESCs (Fig. 1E). Together, our data showed that Sirt2 was up-regulated during mouse ESC differentiation and was not required for mouse ESC self-renewal.

### Knockdown of Sirt2 Promotes Mesoderm and Endoderm Differentiation

**Figure 2:**
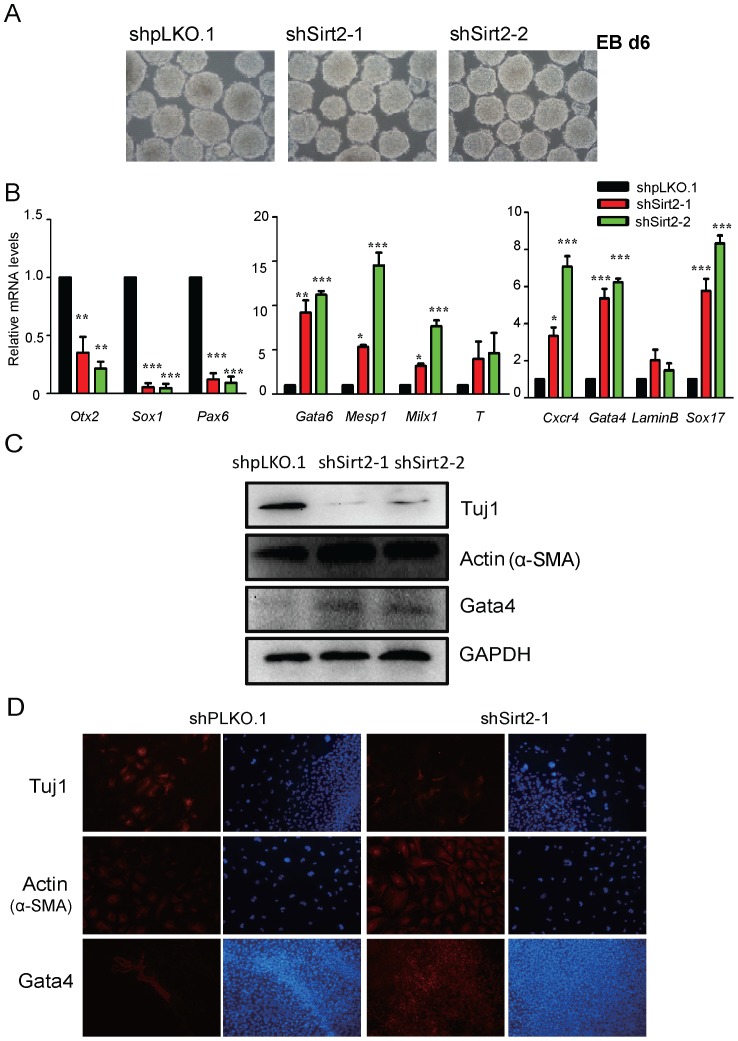
Knockdown of Sirt2 results in the alteration of mouse ESC lineage commitment. (**A**) Phase-contrast images of EB at day 6. All Figures 100×. (**B**) Real-time PCR shows the relative mRNA expression levels of the ectoderm marker genes (Otx2, Sox1 and Pax6), the mesoderm marker genes (Gata6, Mesp2, Mixl1 and T) and the endoderm marker genes (Cxcr4, Gata4, LaminB and Sox17) in control (shpLKO.1) and two Sirt2 stable knockdown cell lines (shSirt2-1 and shSirt2-2) during EB differentiation at day 6. Data were normalized to *Gapdh* mRNA expression levels and are means ±SEM (n = 3). *P*<0.05(*), *P*<0.01(**) and *P*<0.001(***) vs. control EBs generated from shpLKO.1-infected mouse ESCs (One-way ANOVA). (**C**) Western blots to analyze the protein levels of Tuj1, Actin (α-SMA) and Gata4 in EBs generated from control and Sirt2 knockdown ESCs at day 9. GAPDH was used as a protein loading control. (**D**) Immunostaining for Tuj1, Actin (α-SMA) and Gata4 in EBs generated from control and Sirt2 knockdown ESCs at day 9. Cells were counterstained with DAPI (blue). All Figures 100×.

To examine the effect of Sirt2 knockdown on the differentiation of mouse ESCs, we subjected ESCs to EB differentiation and collected samples after 6 days of culture (Fig. 2A). We next used Real-time PCR to survey the expression levels of lineage-specific markers in both control and Sirt2 knockdown EBs (Fig. 2B). Compared to control mouse EBs, in Sirt2 knockdown EBs the expression of ectoderm markers (Otx2, Sox1 and Pax6) was largely abolished, while mesoderm markers (Gata6, Mesp1, Milx1) and ectoderm markers (Cxcr4, Gata4 and Sox17) were expressed at high levels. Western blot analysis showed the ectoderm marker (Tuj1) was suppressed, while the mesoderm (Actin) and ectoderm (Gata4) markers were enhanced in Sirt2 knockdown EBs (Fig. 2C). Immunostaining experiments confirmed this conclusion. The number of Tuj1-positive cells was severely decreased, while Actin-, and GATA4-positive cells were markedly increased in Sirt2 knockdown EBs compared with control EBs (Fig. 2D). Together, these data showed that the down-regulation of Sirt2 expression levels during differentiation altered the formation of all three germ layers.

### GSK3β is Activated in Sirt2 Stable Knockdown Mouse ESCs

**Figure 3:**
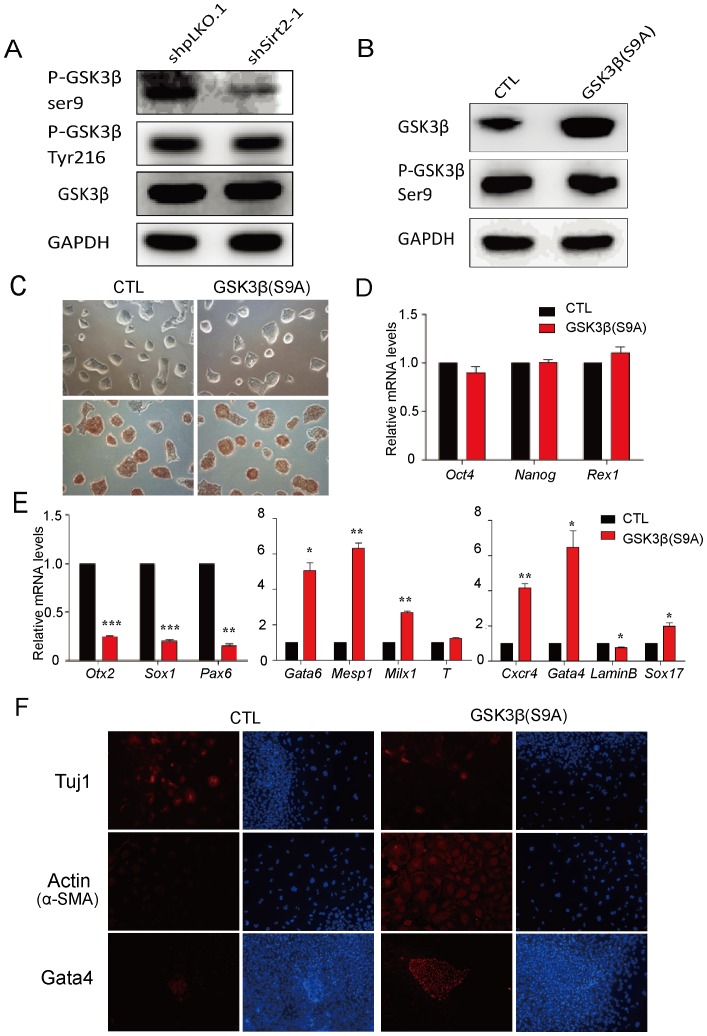
Activated GSK3β is involved in Sirt2 stable knockdown ESC differentiation. (**A**) Western blots analyzing pGSK3β and total GSK3β levels in control (shpLKO.1) and Sirt2 knockdown cells (shSirt2-1) using GAPDH as a loading control. (**B**) The relative protein levels of pGSK3β and total GSK3β levels in GSK3β mutant cell lines. (**C**) AP staining of control and constitutively active GSK3β mutant S9A cell lines. All Figures 100×. (**D**) Real-time PCR analysis for the self-renewal marker genes Oct4, Nanog and Rex1. (**E**) Marker genes of all three germ layers in EBs generated from control and GSK3β-S9A cells at day 6 are verified by real-time PCR. Data were normalized to *Gapdh* mRNA expression levels and are means ±SEM (n = 3). *P*<0.05(*), *P*<0.01(**) and *P*<0.001(***) vs. control EBs generated from FUGW-infected mouse ESCs (Student’s *t*-test). (**F**) Immunofluorescence staining for Tuj1, Actin and Gata4 in EBs generated from control and GSK3β-S9A ESCs at day 9. Cells were counterstained with DAPI (blue). All Figures 100×.

As two independent Sirt2 knockdown cells (shSirt2-1 and shSirt2-2) showed similar expression pattern, we have used shSirt2-1 ESCs for further experiments. We found that GSK3β was activated in Sirt2 stable knockdown cell lines, with a sharp decrease in phosphorylation of Ser9 but not of Tyr216 (Fig. 3A). We then generated a constitutively active GSK3β-S9A mutant mouse ESC line to compare the differentiation phenotype to that of the Sirt2 knockdown cell lines. In the GSK3β-S9A cells, the expression level of GSK3β was greatly increased, but phosphorylation at Ser9 was unchanged (Fig. 3B). The real-time PCR analysis of pluripotent gene expression and alkaline phosphatase staining showed that the self-renewal markers in GSK3β-S9A cells were unchanged compared with control cells (Fig. 3C, D). Subsequent immunostaining confirmed that the expression of the self-renewal marker genes Oct4 and Nanog were not altered in GSK3β-S9A cells (Fig. S1). These data indicated that, similar to the effect of Sirt2 knockdown, activation of GSK3β did not affect the self-renewal properties of mouse ESCs. We then examined whether constitutive activation of GSK3β may cause a similar effect on ESC differentiation as knockdown of Sirt2. During the EB differentiation of GSK3β-S9A cells, the ectoderm marker genes were down-regulated, while the mesoderm and endoderm marker genes were significantly up-regulated; this was similar to what was observed in the Sirt2 knockdown cells (Fig. 3E). Furthermore, the immunostaining results for Tuj1, Actin and Gata4 were consistent with the real-time PCR analysis (Fig. 3F). Thus, we speculate that Sirt2 may play a role in differentiation through activation of GSK3β.

### Lack of GSK3β Blocks the Effects of Sirt2 Knockdown on EB Differentiation

**Figure 4:**
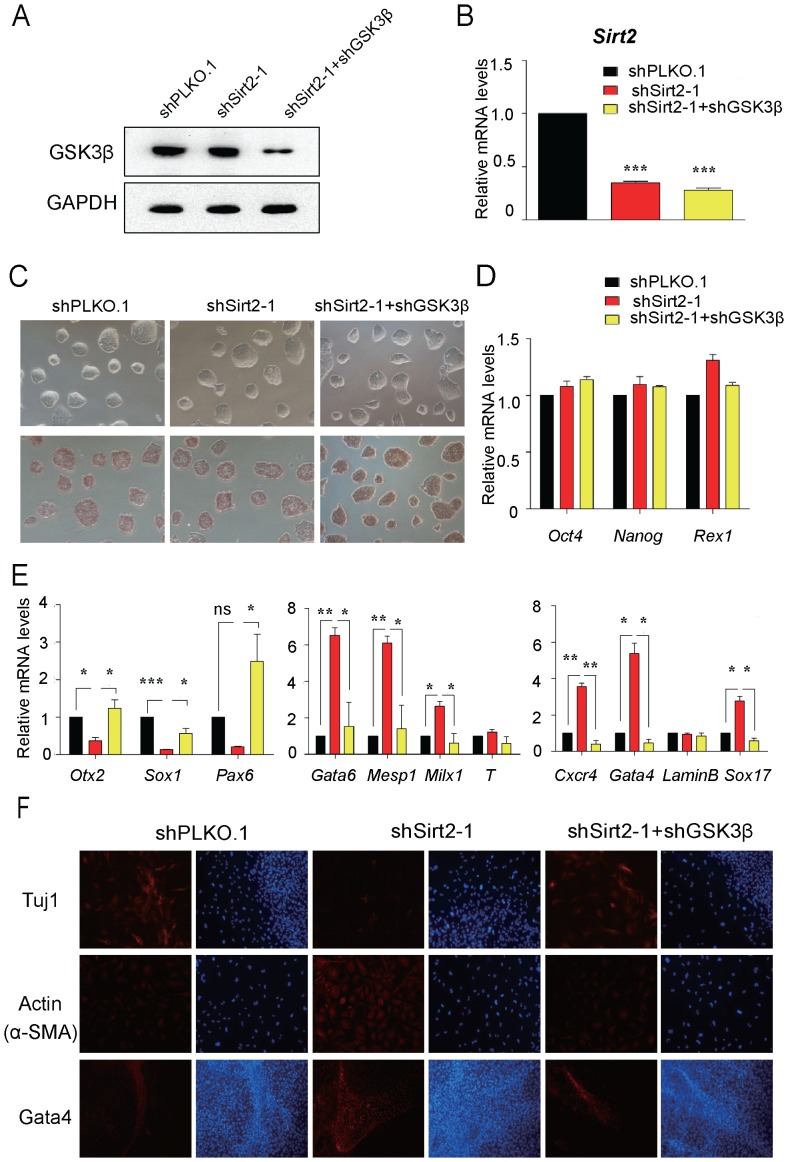
The effect of Sirt2 knockdown on EB differentiation is blocked in the absence of GSK3β. (**A**) Western blots analyzing the extent of the knockdown of GSK3β using GAPDH as a loading control. (**B**) The relative mRNA levels of Sirt2 in three cell lines: control, shsirt2-1, and shsirt2-1+shGSK3β. Data were normalized to *Gapdh* mRNA expression levels and are means ±SEM (n = 3). *P*<0.001(***) vs. control shpLKO.1-infected mouse ESCs (One-way ANOVA). (**C**) AP staining of three cell lines. All Figures 100×. (**D**) The relative mRNA expression levels of the self-renewal marker genes Oct4, Nanog and Rex1. (**E**) The relative mRNA expression levels of the three germ layer marker genes in EB differentiation in three cell lines at day 6. Data were normalized to *Gapdh* mRNA expression levels and are means ±SEM (n = 3). *P*<0.05(*), *P*<0.01(**) and *P*<0.001(***) vs. control EBs generated from shpLKO.1-infected mouse ESCs (One-way ANOVA). (**F**) Immunofluorescence staining for Tuj1, Actin and Gata4 in EBs generated from three cell lines at day 9. Cells were counterstained with DAPI (blue). All Figures 100×.

To test the hypothesis that Sirt2 exerts its effect on differentiation through GSK3β, we performed rescue experiments. Using lentivirus-mediated shRNA knockdown we generated Sirt2 and GSK3β double knockdown cell lines. The protein expression level of GSK3β in the control, shsirt2, and shsirt2+shGSK3β cell lines was verified using western blot analysis (Fig. 4A), and Sirt2 expression levels were obtained using real-time PCR analysis (Fig. 4B). The expression of the self-renewal markers Oct4 and Nanog was examined by real-time PCR analysis, alkaline phosphatase staining (Fig. 4C, D) and immunostaining (Fig. S2), and indicated that the knockdown of Sirt2 and GSK3β did not affect the self-renewal of mouse ESCs. During EB differentiation of the Sirt2 and GSK3β double knockdown cell line, real-time PCR analysis revealed that there was a robust increase in ectoderm marker gene expression, and at the same time, a significant decrease in mesoderm and endoderm marker gene expression, compared with the Sirt2 knockdown cell line (Fig. 4E). The immunostaining experiments showed a similar result compared with the real-time PCR analysis (Fig. 4F). These data confirmed that Sirt2 regulates mouse ESC lineage commitment through GSK3β signaling.

## Discussion

In this study, we describe a new function of Sirt2 in regulating the activity of GSK3β during mouse ESC differentiation and lineage commitment. We found that Sirt2 expression is up-regulated during mouse ESC differentiation and this up-regulation is required for the proper lineage commitment of mouse ESCs. GSK3β is activated when Sirt2 is knocked down in mouse ESCs, and this affects the lineage commitment toward all three germ layers.

The mammalian sirtuin family has many cellular functions, including in cell cycle regulation and cell protection [17]. Previous studies have shown that Sirt1 regulates not only the acetylation levels of histones [13],[18]–[19] but also those of key transcription factors, such as p53, FOXO and p73 [14]. Sirt1 is down-regulated during both human and mouse ESC differentiation. In human ESCs, the expression of neuroectodermal marker genes is increased when Sirt1 is depleted and down-regulated when Sirt1 is over-expressed. Another sirtuin member, Sirt2, is thought to regulate the cell cycle, cellular necrosis and apoptosis [20], but its functions in ESCs are unclear. Our findings show that Sirt2 is up-regulated during the differentiation of mouse ESCs (Fig. 1). Knocking down Sirt2 expression does not affect the self-renewal of mouse ESCs (Fig. 2), however during EB differentiation, the absence of Sirt2 inhibits the formation of ectoderm while promoting the differentiation of mesoderm and endoderm; this reveals that Sirt2 may influence the lineage commitment of mouse ESCs. Histone deacetylases (HDACs) are globular proteins that regulate the acetylation of histone and non-histone proteins. They can be divided into five classes: class I, class IIa, class IIb, class III and class IV. Members of the sirtuin family are also class III HDACs. Previous reports have shown that in mouse ESCs, inhibition of HDACs up-regulate the mRNA and protein levels of Gata4, MEF2C, Nkx2.5, cardiac Actin, and α-SMA, which results in a significant increase in cardiac-lineage commitment through the proteasome pathway [21]. Trichostatin A (TSA), an important HDAC inhibitor, can promote pluripotency in the E14 cell line by increasing the acetylation level of H3K9 [22]. Additional reports have shown that treating ESCs with the HDAC inhibitors TSA and SAHA decreases the expression levels of Oct4 and Nanog and causes a change in global gene expression, leading to early differentiation of ESCs [23]. These findings indicate that deacetylase proteins have an important function in the regulation of ESCs. In addition, because Sirt1 and Sirt2 function differently in ESCs, exploring the roles of other members of the sirtuin family and additional HDAC proteins in ESC differentiation will be useful for our understanding of the regulation of ESC lineage commitment.

Several mechanisms have been proposed to explain how activation of GSK3β plays a role in the differentiation of mouse ESCs. In this study, we identify Sirt2 as a new upstream modulator of GSK3β. Perturbation studies have demonstrated that the lack of Sirt2 dramatically induces the activation of GSK3β through phosphorylation of Ser9. Notably, constitutive activation of GSK3β in ESCs mimics the effect of Sirt2 knockdown during EB differentiation (Fig. 3). Further studies reveal that the expression of marker genes for all three germ layers is rescued when GSK3β is down-regulated (Fig. 4), suggesting that Sirt2 regulates mouse ESC differentiation through GSK3β signaling. Although Sirt2 also regulates other genes during differentiation, these findings suggest that GSK3β may be the key effector through which Sirt2 modulates the lineage commitment of ESCs. Utilizing small-molecule inhibitors, recent studies have shown that GSK3β inhibition may stabilize the naive state of mouse ESCs. ESCs deleted for GSK3β retain normal expression levels of Oct4, Nanog, and Sox2 [24]. In our study, either over-expression of constitutively active GSK3β or deletion of Sirt2 in mouse ESCs preserves a normal expression level of self-renewal marker genes, indicating that activation of GSK3β may not affect the self-renewal of mouse ESCs. Taken together, our data reveal a new cellular function of Sirt2 in controlling ESC differentiation through regulation of GSK3β.

In conclusion, Sirt2 is up-regulated during mouse ESC differentiation. The absence of Sirt2 does not affect self-renewal, but its absence results in altered germ layer differentiation by activating GSK3β through decreased Ser9 phosphorylation. Understanding the mechanisms regulating self-renewal and differentiation will allow the development of new stem cell technologies. Further definition of the role of the sirtuin family in ESCs will provide a new understanding of the mechanisms regulating ESC lineage specification and differentiation, and facilitate the practical application of stem cells.

## Materials and Methods

### Mouse ESC Culture

The mouse ESC line E14.1 were provided by the Cell Bank of Shanghai Institute for Biological Sciences, Chinese Academy of Sciences [25]. Cells were maintained on plates coated with 0.1% gelatin in high glucose Dulbecco’s modified Eagle’s medium (DMEM; Hyclone) containing 15% ES cell qualified fetal bovine serum (Gibco), 1% penicillin and streptomycin (Gibco), 2 mM L-glutamine (Thermo), 100 µM nonessential amino acids (NEAA; Thermo), 100 µM β-mercaptoethanol (Gibco), and leukemia inhibitory factor (LIF; Millipore). For RA-induced differentiation, cells were maintained in culture media without LIF, and 1 µM RA (Sigma) was added.

### EB Formation and in vitro Differentiation Assays of Mouse ESCs

ESCs were harvested by trypsinization (0.25% trypsin) and 2×10^5^ cells were plated on 60-mm petri dishes in 5 ml of mouse ESC culture medium without LIF to generate EBs. RNA was extracted from EBs after 6 days of culture for differentiation analysis using real-time PCR. Primers for real-time PCR are listed in Table S2. For immunostaining analysis of the in vitro differentiation of ESCs, EBs generated as described above were cultured for 3 days and then reseeded onto gelatin-coated 24-well plates for another 6 days.

### Plasmid Construction, Lentiviral Supernatant and Infection of Mouse ESCs

The shRNAs against Sirt2 and GSK3β were cloned into the lentiviral vector pLKO.1 following the pLKO.1-TRC Cloning Vector construction protocol. The lentiviral vector FUGW containing the constitutively active GSK3β mutant S9A was obtained using the QuikChange Lightning Multi Site-Directed Mutagenesis Kit (Agilent Technologies, Cat# 210515-5). All primers are listed in Table S1.

To generate lentivirus, 293T cells were seeded at a density of 4×10^6^ cells per 60-mm dish the day before transfection. The next day, PAX2, VSV-G and the plasmids described above were co-transfected using the Fugene HD transfection reagent (Roche) according to the manufacturer’s protocol. The viral supernatant was harvested at 48 h after transfection and filtered using 0.45 µm Millex-HV (Millipore) filters to remove cell debris. To establish stable cell lines, ES cells were infected with control shRNA lentivirus, Sirt2 shRNA lentivirus, Sirt2+GSK3β shRNA lentivirus, or GSK3β-S9A lentivirus. Forty-eight hours after infection, cells were cultured with medium supplemented with 2 µg/ml puromycin (Sigma) for three passages and expanded. To determine the extent of the knockdown or overexpression, RNA or protein was isolated and real-time PCR or western blotting was performed to determine the expression levels of Sirt2 and GSK3β.

### Reverse-transcription and real-time PCR

Total RNA was extracted from cells using the TRIzol reagent (Invitrogen) according to the manufacturer’s instructions. RNA was reverse-transcribed to cDNA using the TIANScript RT Kit (TIANGEN) following the manufacturer’s instructions. Real-time PCR was performed for 40 cycles using the Takara Ex Taq PCR kit (Takara) in a Stratagene MX3000P QPCR system (Stratagene). The relative expression values were normalized to *Gapdh*. The primer sequences used are listed in Table S2.

### Western Blots

Protein from cultured cells was extracted, separated on reduced SDS-PAGE gels, and transferred to nitrocellulose membranes. The following primary antibodies were used: anti-Sirt2 (Millipore), anti-GSK3β (Cell Signaling Technologies), anti-GSK3β (Phospho-Ser9), anti-GSK3β (Phospho-Tyr216) (both from Signalway Antibody), and anti-GAPDH (Santa Cruz). The blots were analyzed using an ImageQuant LAS 4000 mini imager (GE Healthcare Life Science).

### Alkaline Phosphatase (AP) Staining and Immunostaining

For AP staining, the FastRed Alkaline Phosphatase Kit (Sigma) was used according to the manufacturer’s protocol. For immunostaining, cells were washed twice with PBS and fixed with 4% paraformaldehyde in PBS for 20 min. Fixed cells were permeabilized with 0.2% Triton X-100 for 5 min. Cells were then blocked in 10% FBS in PBS for 1 h. Cells were stained with primary antibody (diluted 1∶1000 in 10% FBS in PBS) overnight at 4°C, and then washed three times with 10% FBS in PBS. Cells were stained with secondary antibody (1∶1000) for 45 min in the dark at room temperature. Anti-Oct4 (Santa Cruz), anti-Nanog (Abcam), anti-Tuj1 (Covance), anti-GATA4 (Santa Cruz), and anti-Actin (α-SMA) (Sigma) antibodies were used for immunostaining. The cells were counterstained with DAPI. The cells were examined under fluorescence microscopy to capture images.

### Data Analysis

For data analysis, GraphPad Prism 4.0 software was used. The data were calculated as the means ± SEM for three independent experiments. Comparisons between means were performed by one-way analysis of variance (ANOVA) for multiple (>2) groups or Student’s *t*-test for comparing two means of independent samples. Differences were considered to be significant at *P*<0.05(*), *P*<0.01(**) and *P*<0.001(***).

## Supporting Information

Figure S1 **Immunofluorescence staining for Oct4 and Nanog in control and GSK3β mutant S9A cell lines.** Cells were counterstained with DAPI (blue). All Figures 100×.
(TIF)

Figure S2 **Immunofluorescence staining for Oct4 and Nanog in three cell lines: control, shsirt2, and shsirt2+shGSK3β. Cells were counterstained with DAPI (blue).** All Figures 100×.
(TIF)

Table S1 Primers used for plasmids construction.
(DOC)

Table S2 Primers used for quantitative real-time PCR.
(DOC)
