# Supplementary figures and images for: Activation of GSK3β by Sirt2 Is Required for Early Lineage Commitment of Mouse Embryonic Stem Cell
Source: PLoS One. 2013 Oct 18;8(10):e76699. doi: 10.1371/journal.pone.0076699 (PMC3800056; doi:10.1371/journal.pone.0076699)

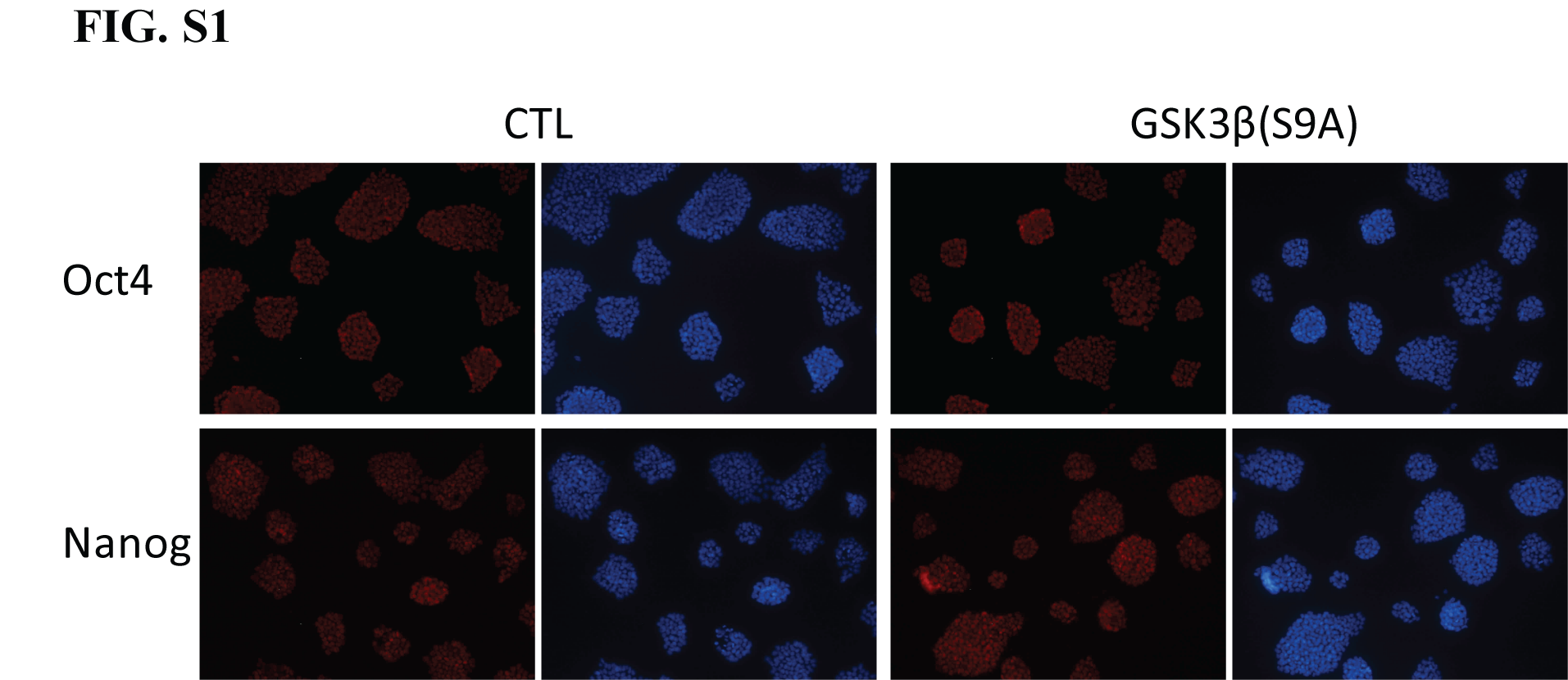

Supplement: Figure S1 — Immunofluorescence staining for Oct4 and Nanog in control and GSK3β mutant S9A cell lines. Cells were counterstained with DAPI (blue). All Figures 100×. (TIF) [file pone.0076699.s001.tif]

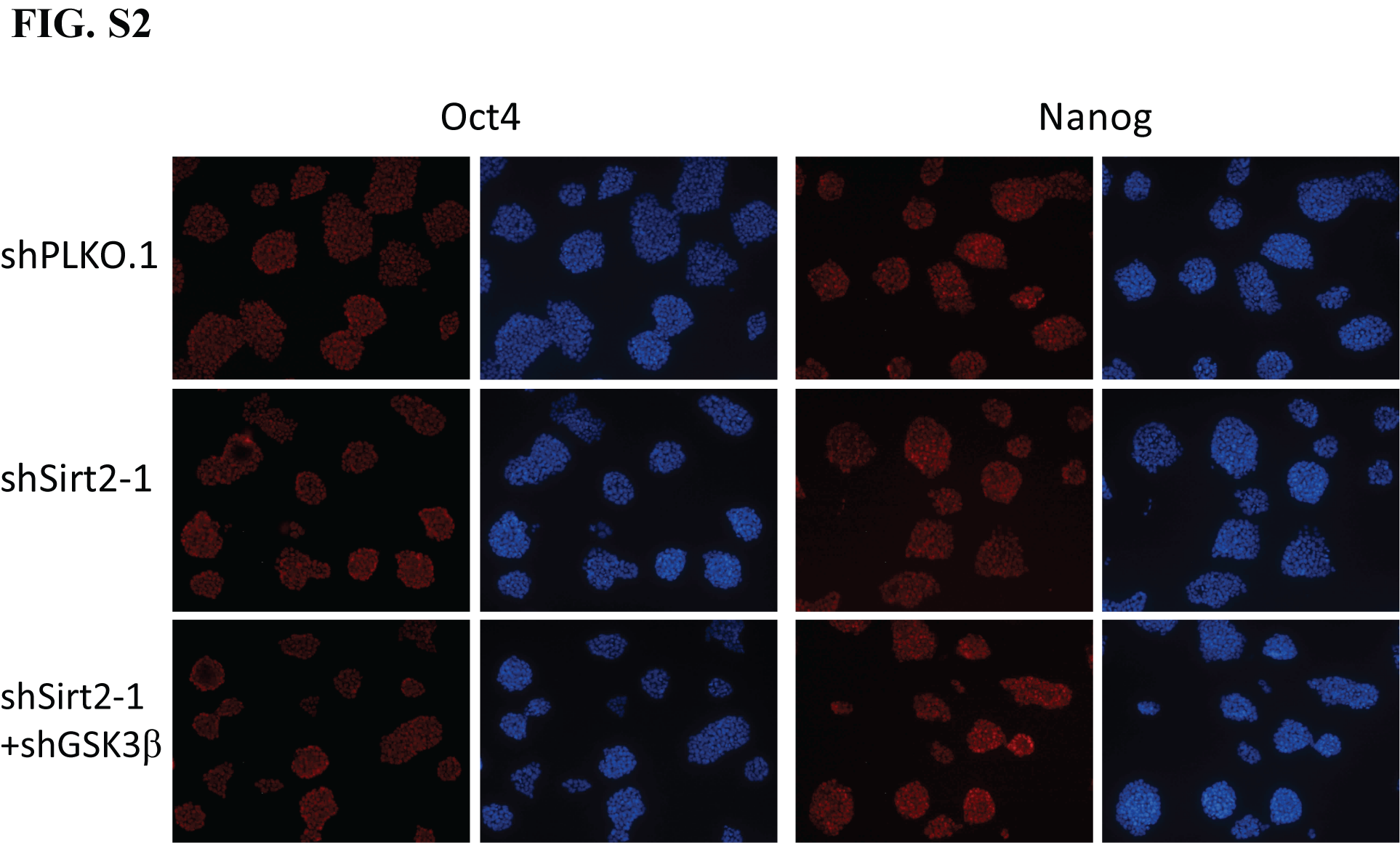

Supplement: Figure S2 — Immunofluorescence staining for Oct4 and Nanog in three cell lines: control, shsirt2, and shsirt2+shGSK3β. Cells were counterstained with DAPI (blue). All Figures 100×. (TIF) [file pone.0076699.s002.tif]
