# Supplementary material for: Activation of GSK3β by Sirt2 Is Required for Early Lineage Commitment of Mouse Embryonic Stem Cell
Source: PLoS One. 2013 Oct 18;8(10):e76699. doi: 10.1371/journal.pone.0076699 (PMC3800056; doi:10.1371/journal.pone.0076699)
Supplement: Table S1 — Primers used for plasmids construction. (DOC) [file pone.0076699.s003.doc]

**Table S1. Primers used for plasmids construction**

| Primer |  | Sequence (5’ to 3’) |
| --- | --- | --- |
| *shSirt2-1* | *Forward*  *Reverse* | CCGGTTGACTCCAAGAAGGCCTACACTCGAGTGTAGGCCTTCTTGGAGTCAATTTTTG  AATTCAAAAATTGACTCCAAGAAGGCCTACACTCGAGTGTAGGCCTTCTTGGAGTCAA |
| *shSirt2-2* | *Forward*  *Reverse* | CCGGCAGTGTCAGAGTGTGGTAAAGCTCGAGCTTTACCACACTCTGACACTGTTTTTG  AATTCAAAAACAGTGTCAGAGTGTGGTAAAGCTCGAGCTTTACCACACTCTGACACTG |
| *shGSK3β* | *Forward*  *Reverse* | CCGGCGGGACCCAAATGTCAAACTACTCGAGTAGTTTGACATTTGGGTCCCG TTTTTG  AATTCAAAAACGGGACCCAAATGTCAAACTACTCGAG TAGTTTGACATTTGGGTCCCG |
| *GSK3β(S9A)* | *Forward*  *Reverse* | CACAGACCGGTATGTCGGGGCGACCGAGAACCACCGCCTTTGC  CACAGACCGGTTCAGGTGGAGTTGGAAGCTGATGC |
