# Supplementary material for: Activation of GSK3β by Sirt2 Is Required for Early Lineage Commitment of Mouse Embryonic Stem Cell
Source: PLoS One. 2013 Oct 18;8(10):e76699. doi: 10.1371/journal.pone.0076699 (PMC3800056; doi:10.1371/journal.pone.0076699)
Supplement: Table S2 — Primers used for quantitative real-time PCR. (DOC) [file pone.0076699.s004.doc]

**Table S2. Primers used for quantitative real-time PCR**

| Primer |  | Sequence ( 5’ to 3’) |
| --- | --- | --- |
| *Oct4* | *Forward*  *Reverse* | CACGAGTGGAAAGCAACTCA  AGATGGTGGTCTGGCTGAAC |
| *Nanog* | *Forward*  *Reverse* | ACCTGAGCTATAAGCAGGTTAAGAC  GTGCTGAGCCCTTCTGAATCAGAC |
| *Rex1* | *Forward*  *Reverse* | GATTCACATCCTAACCCACGCA  TATCCCCAGTGCCTCTGTCATT |
| *Otx2* | *Forward*  *Reverse* | CCATGACCTATACTCAGGCTTCAGG  GAAGCTCCATATCCCTGGGTGGAAAG |
| *Sox1* | *Forward*  *Reverse* | TTACTTCCCGCCAGCTCTTC  TGATGCATTTTGGGGGTATCTCTC |
| *Pax6* | *Forward*  *Reverse* | AACCTGGCTAGCGAAAAGCA  CCCGTTCAACATCCTTAGTTTATC |
| *Gata6* | *Forward*  *Reverse* | GACGGCACCGGTCATTACC  ACAGTTGGCACAGGACAGTCC |
| *Mesp1* | *Forward*  *Reverse* | GTTCCTGTACGCAGAAACAGCATC  CAAGGAGGGTTGGAATGGTACAGT |
| *Mixl1* | *Forward*  *Reverse* | AGTTGCTGGAGCTCGTCTTC  AGGGCAATGGAGGAAAACTC |
| *T* | *Forward*  *Reverse* | GGTGGCTTGTTCCTGGTGC  GTAGGTGGGCTGGCGTTAT |
| *Cxcr4* | *Forward*  *Reverse* | TCCAACAAGGAACCCTGCTTC  TTGCCGACTATGCCAGTCAAG |
| *Gata4* | *Forward*  *Reverse* | CCCTACCCAGCCTACATGG  ACATATCGAGATTGGGGTGTCT |
| *LaminB* | *Forward*  *Reverse* | CCCCAATCTCTGTGAACCATG  GCAATTTGCACCGACACTGA |
| *Sox17* | *Forward*  *Reverse* | CGAGCCAAAGCGGAGTCTC  TGCCAAGGTCAACGCCTTC |
| *Gapdh* | *Forward*  *Reverse* | AGGTCGGTGTGAACGGATTTG  TGTAGACCATGTAGTTGAGGTCA |
| *Sirt2* | *Forward*  *Reverse* | AGCCAACCATCTGCCACTAC  CCAGCCCATCGTGTATTCTT |
| *Sirt1* | *Forward*  *Reverse* | AGAACCACCAAAGCGGAAA  TCCCACAGGAGACAGAAACC |
| *Sirt3* | *Forward*  *Reverse* | CTACATGCACGGTCTGTCGAA  GCCAAAGCGAAGTCAGCCATA |
